# Supplementary material for: Global transcontinental power pools for low-carbon electricity
Source: Nat Commun. 2023 Dec 15;14:8350. doi: 10.1038/s41467-023-43723-z (PMC10724180; doi:10.1038/s41467-023-43723-z)
Supplement: Supplementary file 1 — Supplementary information [file 41467_2023_43723_MOESM1_ESM.pdf]

Supplementary Materials for
Global transcontinental power pools for low-carbon electricity

Haozhe Yang[1], Ranjit Deshmukh[1,2], Sangwon Suh[1,*]

[1] Bren School of Environmental Science and Management, University of California, Santa Barbara, CA, USA

[2] Environmental Studies Program, University of California, Santa Barbara, CA, USA

Corresponding author: suh@bren.ucsb.edu

Content

1. Supplementary Figures 1-7

2. Supplementary Tables 1-12

3. Supplementary Data

Supplementary Figures

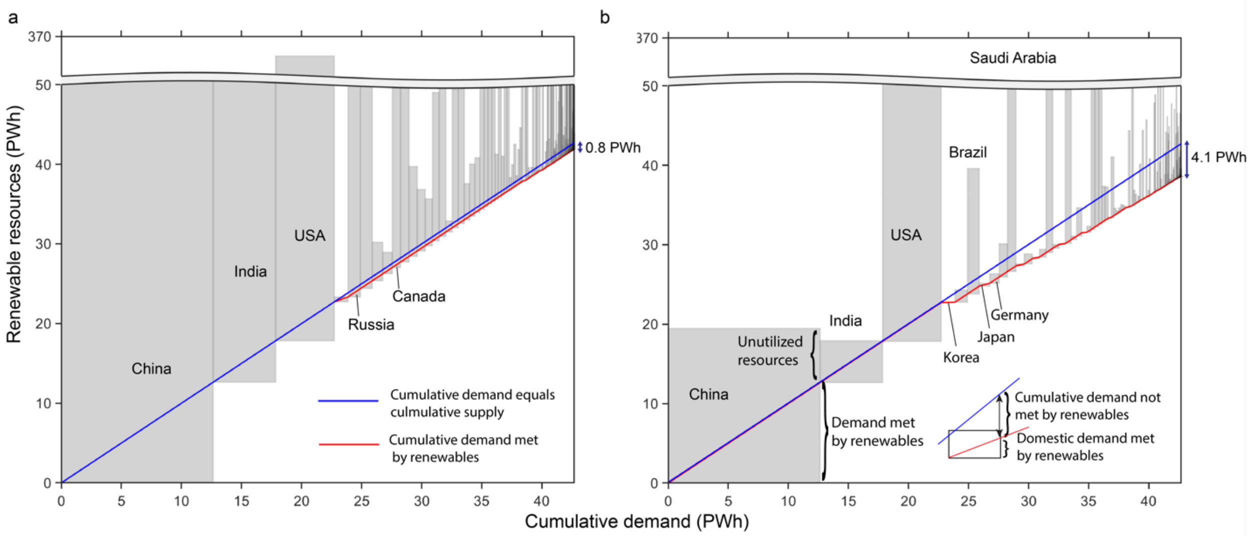

Supplementary Figure 1. Supply and demand of renewable electricity in 2050 without considering the temporal variation of renewable energy.

Renewable potential and electricity demand in 2050 by country under the country scenario using **a** all suitable sites for renewables and **b** top 10% of suitable sites at the global level. In **a** and **b,** each rectangle represents the demand for electricity (horizontal dimension) and the available renewable resources potential (vertical dimension). The blue diagonal line represents demand equals renewable electricity potential by country ($y = x$ line). The red line represents the cumulative demand met by renewable potential by country, without considering the temporal variation of renewables. Within a country's rectangle, when the slope of the red line is smaller than the slope of the blue line, country-level electricity demand is infeasible to be met only by renewable potential.

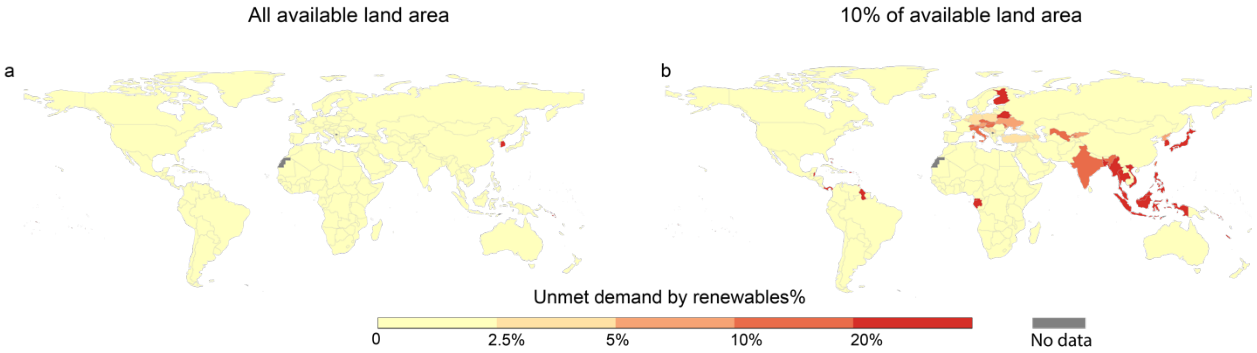

Supplementary Figure 2. Unmet demand with only the supply of renewable resources under the country scenario after adding existing transmission lines in Europe.
a all available sites for renewables and **b** top 10% suitable sites at the global level.

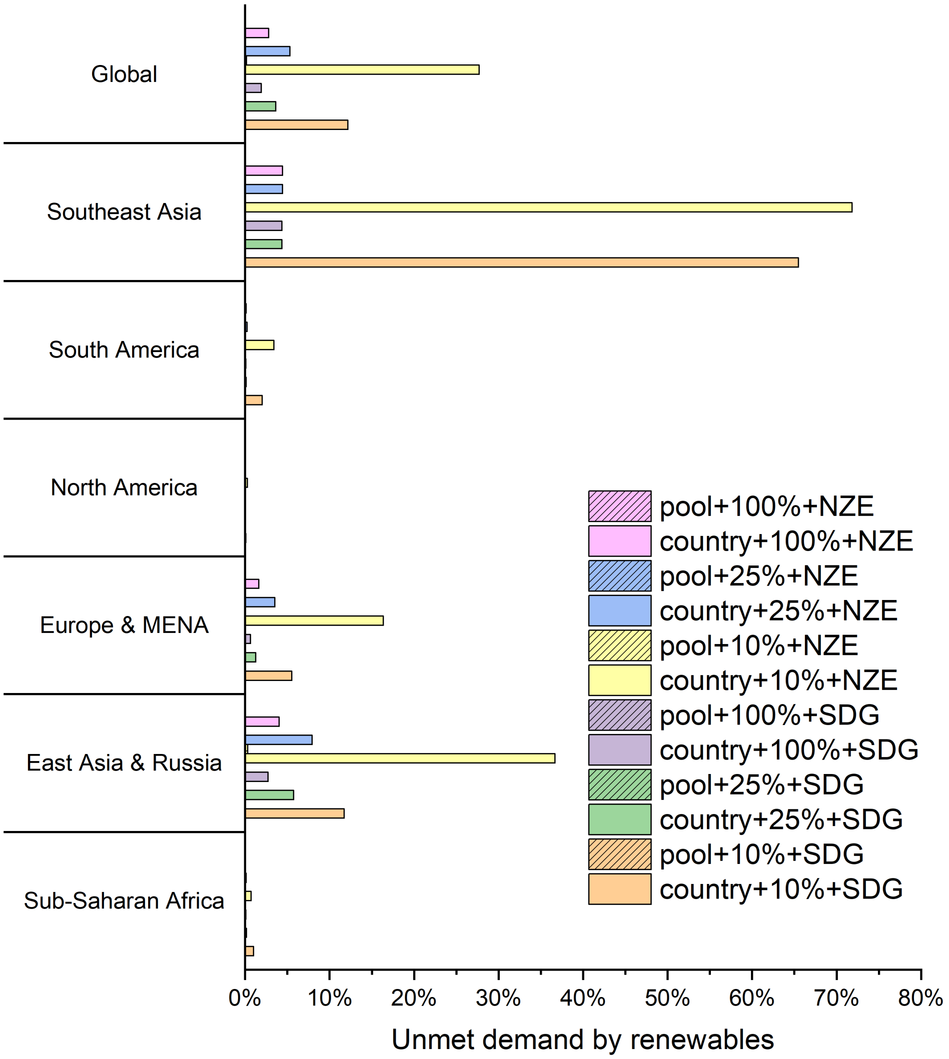

Supplementary Figure 3. Unmet demand by renewable electricity generation under different scenarios.

‘country’ and ‘pool’ refer to the country and transcontinental scenario. ‘10%’, ‘25%’ and 100% refer to the top 10% sites, top 25% sites and all available sites for renewables. SDG and NZE refer to the demand scenarios under the UN Sustainable Development Goals and the IEA Net Zero Emissions by 2050, respectively.

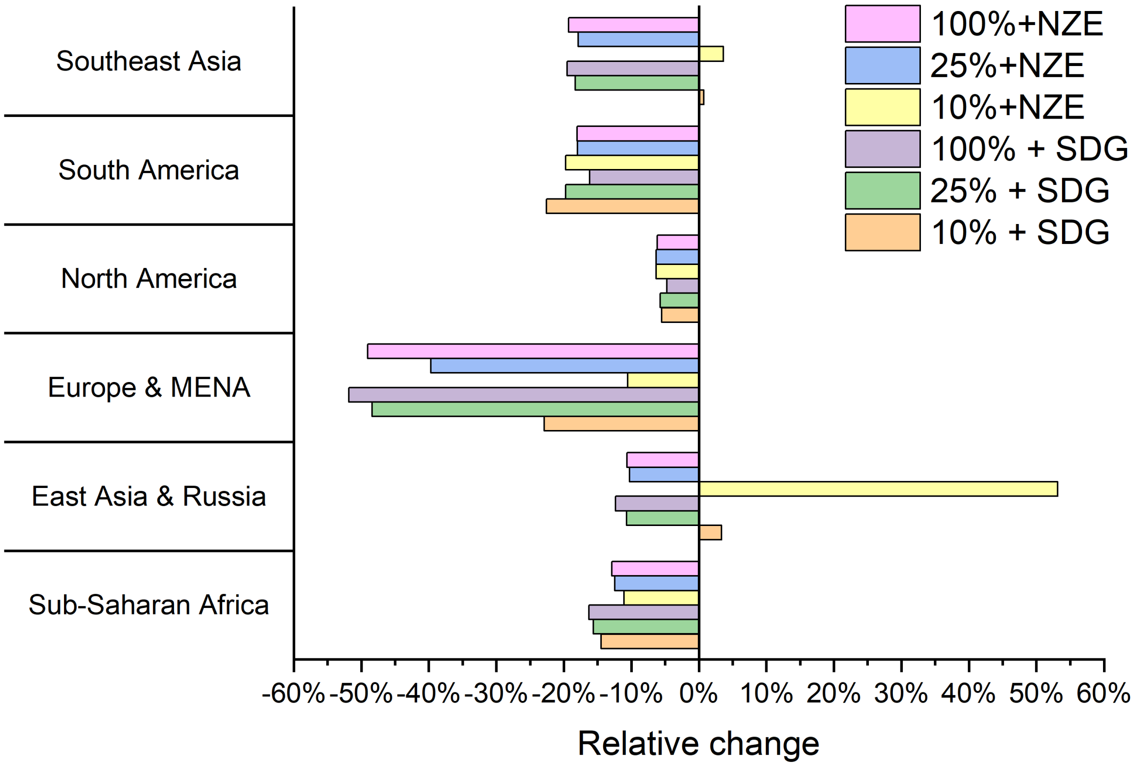

Supplementary Figure 4. Cost change ($/MWh) by transcontinental power pools under different scenarios compared to the country scenario.

'10%', '25%' and 100% refer to the top 10% sites, the top 25% sites and all suitable sites for renewables. SDG and NZE refer to the demand scenarios.

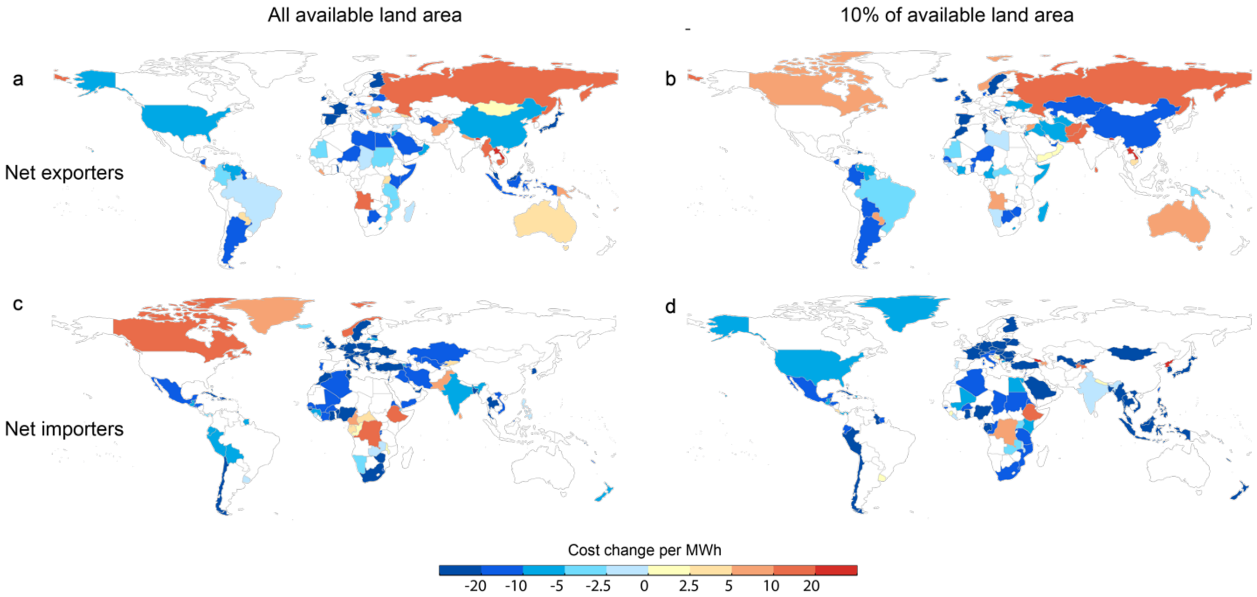

Supplementary Figure 5. Change in system costs without the cost of transmission under the transcontinental scenario compared to the country scenario.

Change of system costs in net exporters assuming **a** all suitable sites for renewables, and **b** global top 10% suitable sites. Change of system costs in net importers assuming **c** all available sites for renewables, and **d** top 10% suitable sites.

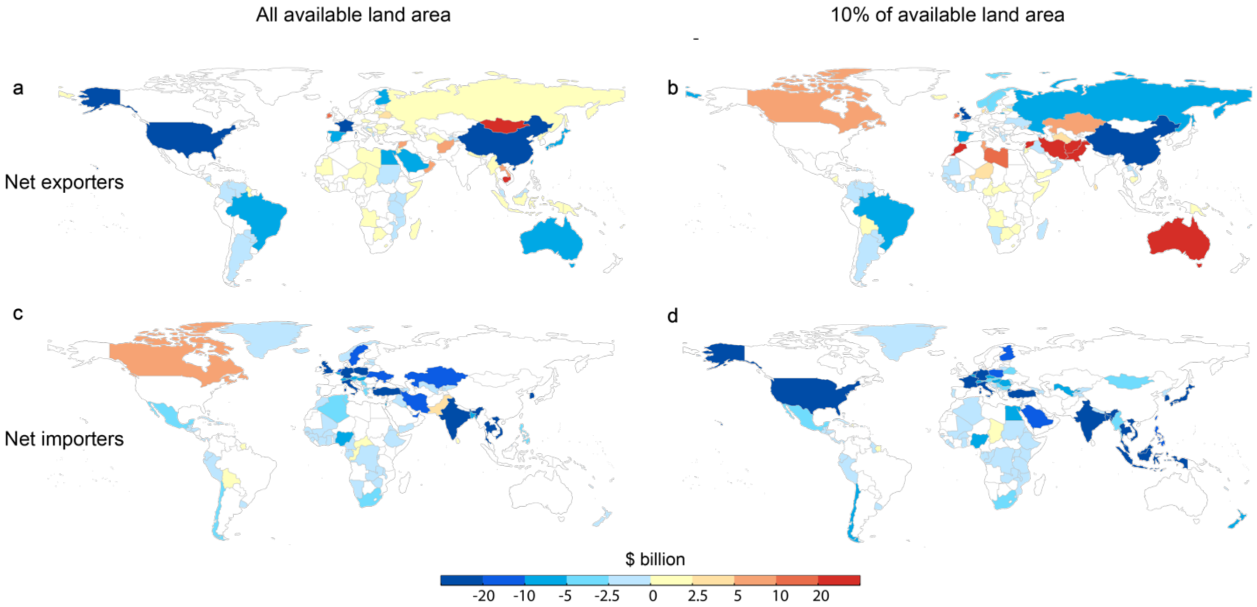

Supplementary Figure 6. Change in investment costs within the boundary under the transcontinental scenario compared to the country scenario.

Change of investment cost in net exporters assuming **a** all suitable sites for renewables, and **b** global top 10% suitable sites. Change of investment cost in net importers assuming **c** all suitable sites for renewables, and **d** top 10% suitable sites.

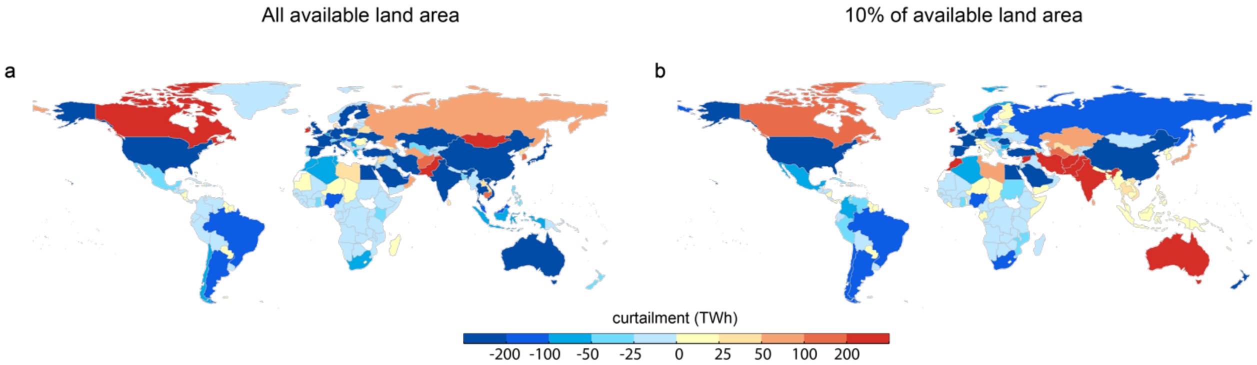

Supplementary Figure 7. Change of curtailment (TWh) by transcontinental power pools under different demand scenarios compared to the country scenario.

Change of curtailment in net exporters assuming **a** all suitable sites for renewables, and **b** global top 10% suitable sites. Change of system costs in net importers assuming **c** all available sites for renewables, and **d** top 10% suitable sites.

Supplementary Tables

Supplementary Table 1. Unmet demand (PWh) in Europe & MENA under the country scenario, with and without existing transmission lines.

Sites	No transmission lines	Existing transmission lines
10%	0.4 (5%)	0.3 (4%)
All	0.05 (0.7%)	0.03 (0.4%)

Supplementary Table 2. System cost ($/MWh) in Europe & MENA under the country scenario with and without existing transmission lines, and the system cost under the 'transcontinental' scenario.

Sites	No transmission lines	Existing transmission lines	Power pool
10%	77	75	59
All	91	81	44

Supplementary Table 3. The unmet demand using the capacity expansion model with a 3-hour temporal resolution model, and the validation using an 8760-hour operation model. The 2050 global demand is 43 PWh under the SDG (sustainable development goal) scenario.

Scenario	Model	Top 10% sites + SDG	Top 100% sites + SDG
Country	3-hour(1)	12%	1.9%
	8760(2)	12%	1.9%
Transcontinental	3-hour	0	0
	8760	0	0

(1) Optimized the capacity investment by using 3-hour temporal resolution in a whole year,

(2) Simulated the operation with fixed capacities across 8760 hours.

Supplementary Table 4. The unmet demand using the capacity expansion model with 24 representative days, and the validation using an 8760-hour operation model. The 2050 global demand is 43 PWh under the SDG scenario.

Scenario	Model	Top 10% sites + SDG	Top 100% sites + SDG
Country	Reduced(1)	12%	1.9%
	8760(2)	12%	2.2%
Transcontinental	Reduced	0	0
	8760	0.7%	0.9%

(1) Optimized the capacity investment by using 24 representative days (576 hours) within a year,

(2) Simulated the operation with fixed capacities across 8760 hours.

Supplementary Table 5. Change of the system cost in transcontinental power pools compared with the case without power pool by using different temporal resolutions in the capacity expansion model.

Pool	100% land availability		10% land availability	
	24 days	3-hour	24 days	3-hour
Sub-Saharan Africa	-14%	-16%	-11%	-14%
East Asia & Russia	-13%	-12%	-5.6%	3.3%
Europe & Middle East	-44%	-52%	-32%	-23%
North America	-2.9%	-4.7%	-3.4%	-5.5%
South America	-13%	-16%	-19%	-23%
Southeast Asia & Oceania	-15%	-19%	-4.4%	0.7%

Supplementary Table 6. Cost parameters for renewable energy technology in 2020.

	PV power plant	Rooftop PV	CSP-no storage	Onshore wind	Offshore wind	Hydropower
Land use factor (MW/km^2)	37[1] (31×1.17)	12[2]	15[1]	3[3]	3[3]	
Capital cost ($/kW)	883[4]	1817[(1)5]	3907[(2)5]	1355[4]	3185[4]	1870[4]
O&M cost[5] ($/kW)	23	29	66	43	109	30
Variable cost[5] ($/MWh)	0	0	3.5			

(1) The capital cost for utility-scale and rooftop PV is $1333/kW and $2734/kW from NREL. The global average capital cost for rooftop PV is calculated as 883·2734/1333=1817.

(2) The capital cost for CSP-no storage=Turbine cost + 1.2 · field cost.

Supplementary Table 7. Assumptions for the rooftop PV

Parameter	Value
Ratio of urban area[8]	0.25
Share of suitable rooftop[9]	0.33

Supplementary Table 8. Cost parameters for the storage technology[5] in 2020.

	Battery storage	Pumped hydro
Capital cost ($/kW)	249	1999
Energy cost ($/kWh)	369	NA
O&M cost ($/kW)	6	18
O&M cost ($/kWh)	9	NA
Variable cost ($/MWh)	0	0.5125
Roundtrip efficiency	85%	80%

Supplementary Table 9. Cost parameters for the HDVC[6].

	HVDC
Capital cost ($/(km·kW))	1.044
O&M cost (($/(km·kW))	0.003
Lifetime (years)	50
Transmission loss (%/1000 km)	1.6
Converter capital cost ($/kW)	180
Converter O&M ($/kW)	1.8
Converter pair loss (%)	1.4

Supplementary Table 10. Cost projection for capital cost from 2030-2050[5].

Technology	2020	2030	2040	2050
Battery storage capacity	1.00	1.06	0.93	0.80
Battery storage energy	1.00	0.43	0.37	0.32
PV	1.00	0.56	0.51	0.46
Rooftop	1.00	0.37	0.33	0.29
CSP	1.00	0.68	0.59	0.57
Wind	1.00	0.65	0.58	0.52
Offshore	1.00	0.73	0.66	0.62
Pumped hydro	1.00	1.00	1.00	1.00
Hydropower	1.00	1.00	1.00	1.00
HVDC	1.00	1.00	1.00	1.00

Supplementary Table 11. Cost projection for O&M cost from 2030-2050[5].

Technology	2020	2030	2040	2050
Battery storage capacity	1.00	1.06	0.93	0.80
Battery storage energy	1.00	0.43	0.37	0.32
PV	1.00	0.67	0.63	0.59
Rooftop	1.00	0.45	0.42	0.38
CSP	1.00	0.86	0.85	0.85
Wind	1.00	0.91	0.84	0.77
Offshore	1.00	0.77	0.69	0.63
Pumped hydro	1.00	1.00	1.00	1.00
Hydro	1.00	1.00	0.96	0.96
HVDC	1.00	1.00	1.00	1.00

Supplementary Table 12. Growth rates of electricity demand under SDG (Sustainable development goal) [10] and NZE[11] (Net zero emission) scenarios.

	SDG	NZE
North America	0.7%	2.3%
United States	0.6%	2.3%
Central & South America	2.0%	4.7%
Brazil	1.7%	4.7%

Europe	1.2%	2.3%
European Union	1.1%	2.3%
Africa	4.1%	4.7%
South Africa	0.8%	4.7%
Middle East	2.4%	4.7%
Eurasia	0.5%	4.7%
Russia	0.4%	4.7%
Asia Pacific	2.4%	4.7%
China	2.1%	4.7%
India	4.5%	4.7%
Japan	-0.2%	2.3%
Southeast Asia	3.2%	4.7%

Reference

1. Ong, S., Campbell, C., Denholm, P., Margolis, R. & Heath, G. *Land-use requirements for solar power plants in the United States*. (2013).

2. Gagnon, P., Margolis, R., Melius, J., Phillips, C. & Elmore, R. *Rooftop solar photovoltaic technical potential in the United States. A detailed assessment*. (2016).

3. Denholm, P., Hand, M., Jackson, M. & Ong, S. *Land use requirements of modern wind power plants in the United States*. (2009).

4. International Renewable Energy Agency. Renewable Power Generation Costs in 2020. /publications/2021/Jun/Renewable-Power-Costs-in-2020 https://www.irena.org/publications/2021/Jun/Renewable-Power-Costs-in-2020.

5. Data | Electricity | 2022 | ATB | NREL. https://atb.nrel.gov/electricity/2022/data.

6. Bogdanov, D. et al. Radical transformation pathway towards sustainable electricity via evolutionary steps. Nature Communications 10, 1077 (2019).

7. Data | Electricity | ATB | NREL. https://atb-archive.nrel.gov/electricity/2020/data.php.

8. Akbari, H., Menon, S. & Rosenfeld, A. Global cooling: increasing world-wide urban albedos to offset CO2. Climatic Change 94, 275–286 (2009).

9. Deng, Y. Y. et al. Quantifying a realistic, worldwide wind and solar electricity supply. Global Environmental Change 31, 239–252 (2015).

10. IEA. World Energy Outlook 2019. https://www.iea.org/reports/world-energy-outlook-2019 (2019).
11. IEA. World Energy Outlook 2022. IEA https://www.iea.org/reports/world-energy-outlook-2022 (2022).
