# Supplementary material for: Global transcontinental power pools for low-carbon electricity
Source: Nat Commun. 2023 Dec 15;14:8350. doi: 10.1038/s41467-023-43723-z (PMC10724180; doi:10.1038/s41467-023-43723-z)
Supplement: Supplementary file 3 — Description of Additional Supplementary Files [file 41467_2023_43723_MOESM3_ESM.pdf]

Description of Additional Supplementary Files

File Name: Supplementary Data 1
Description: Transmission lines in power pools. “tx_new_lin” represents new transmission lines. “tx_spec” represents existing transmission lines. We assume the transmission lines are bidirectional.
